# Association Between Chronic Obstructive Pulmonary Disease Severity and Heart Failure in a Tertiary Care Cohort

**DOI:** 10.7759/cureus.108941

**Published:** 2026-05-15

**Authors:** Noor Ul Ann Malik, Julianne F Powell, Junaid Muzaffar, Khawaja Abdullah Zahid, Chinedum S Iheukwumere, Muhammad Tayyab, Syed Muzammil Hussain Shah

**Affiliations:** 1 Emergency Medicine, Family Hospital Zafarwal, Narowal, PAK; 2 Medicine, Sheffield Teaching Hospitals NHS Foundation Trust, Sheffield, GBR; 3 Cardiology, Dr. Faisal Masood Teaching Hospital, Sargodha, Sargodha, PAK; 4 Cardiology, Bahauddin Cardiac Complex, Mandi Bahauddin, PAK; 5 General Internal Medicine, York and Scarborough Teaching Hospitals NHS Foundation Trust, Scarborough, GBR; 6 Cardiology, Azra Naheed Medical College, Lahore, PAK; 7 Emergency Medicine, Nawaz Sharif Social Security Teaching Hospital, Lahore, Lahore, PAK

**Keywords:** chronic obstructive pulmonary disease (copd), gold classification, heart failure, hypertension, patients

## Abstract

Background: Chronic obstructive pulmonary disease (COPD) is a progressive respiratory disorder that often coexists with cardiovascular comorbidities, particularly heart failure (HF).

Objective: To evaluate the association between the severity of COPD and the prevalence of HF in patients treated at a tertiary care hospital.

Methodology: This prospective cross-sectional analytical study was conducted at Dr. Faisal Masood Teaching Hospital, Sargodha, Pakistan, from July 2024 to July 2025 on 225 patients with confirmed COPD. The severity of COPD was classified according to the Global Initiative for Chronic Obstructive Lung Disease (GOLD) criteria. Patients were evaluated for HF through clinical examination, echocardiographic assessment, and biomarker analysis, where available.

Results: The mean age of participants was 61.4 ± 9.7 years, with a male predominance (69.3%). Overall, 72 patients (32.0%) were diagnosed with HF. The prevalence of HF increased progressively with COPD severity: 5.9% in GOLD I, 17.7% in GOLD II, 42.7% in GOLD III, and 70.0% in GOLD IV (p < 0.001). On multivariate analysis, severe-to-very-severe COPD (OR = 5.4, 95% CI: 2.8-10.5, p < 0.001), hypertension (OR = 2.7, 95% CI: 1.4-5.3, p = 0.002), and age ≥ 60 years (OR = 1.9, 95% CI: 1.1-3.8, p = 0.04) were independent predictors of HF.

Conclusion: It is concluded that increasing COPD severity is significantly associated with a higher incidence of HF. Patients with advanced COPD are at greater risk of developing cardiac dysfunction due to overlapping pathophysiological mechanisms and shared risk factors.

## Introduction

Chronic obstructive pulmonary disease (COPD) continues to remain a worldwide health challenge. Defined as a condition with chronic, pervasive limitations and progressive declines, it causes emphysema and chronic bronchitis to further deteriorate pulmonary capabilities and, to an extent, compromise the quality of life [1]. WHO statistics indicate that the impact of the condition stretches to over 300 million individuals, as it remains the third leading cause of mortality. As the advancements in the diagnosis and treatment of the condition continue to improve, the burden of the condition is high, particularly in developing countries, where the burden of smoking and exposure to high indoor pollution is evident [2]. In these regions, advanced disease is often presented with multiple and complicated comorbidities as well as limited specialized care [3]. In the last couple of decades, the predominant nature of the condition has also expanded its range to include systemic effects across COPD [4]. The systemic impact of the condition is attributed to chronic, low-grade systemic inflammation, which in turn has an impact on the vasculature and the endothelium. Of all of them, the most significant and debilitating impact is heart failure (HF) [5,6].

The intersection of COPD and HF poses numerous diagnostic and treatment challenges due to overlapping symptoms of each disease, including dyspnea, cough, and exercise tolerance. The overlapping symptoms can cause misdiagnosis and lead to delays in identifying HF, which can result in adverse outcomes and increased mortality risk [6]. Several pathophysiological factors explain the association between COPD and femoral head fractures (FHF). Severe forms of COPD with chronic hypoxia and hypercapnia can cause pulmonary vasoconstriction and remodeling of the pulmonary vasculature, which in turn leads to pulmonary hypertension [7]. The right ventricle bears the consequences of these hemodynamic changes and can lead to right-sided HF or cor pulmonale. The consequences of systemic inflammation observed in chronic COPD are the elevation of peripheral inflammatory cytokines (e.g., interleukin-6 (IL-6), tumor necrosis factor-alpha (TNF-α), C-reactive protein (CRP)), which lead to adverse cardiac remodeling and myocardial function [8].

Other consequences of advanced COPD, including oxidative stress, endothelial dysfunction, and platelet activation, aggravate the cardiovascular disease risks in these patients. This increased risk of developing HF in patients with COPD is well documented in the literature [9]. Research on a large population-based cohort showed that individuals with advanced stages of COPD have an incidence of HF that is two to three times higher than individuals without COPD, and this is independent of other traditional risk variables, including hypertension, diabetes, and coronary artery disease (CAD) [10]. This association is driven not only by shared causes, such as smoking and age, but by the systemic impact of chronic lung disease as well [11]. The systemic impact of chronic lung disease must not be ignored, but neither must the reverse impact of HF; patients with HF may have pulmonary congestion that profoundly decreases lung function, potentially simulating or worsening the symptoms of COPD [12].

The main objective of the study is to evaluate the association between the severity of COPD and the prevalence of HF in patients treated at a tertiary care hospital.

## Materials and methods

This prospective cross-sectional analytical study was conducted at Dr. Faisal Masood Teaching Hospital, Sargodha, Pakistan, from July 2024 to July 2025 on 225 patients with confirmed COPD. Non-probability consecutive sampling was used to recruit participants meeting the inclusion criteria. This study was conducted after the approval of the Institutional Review Board (IRB) of Dr. Faisal Masood Teaching Hospital, Sargodha (approval 497/DFMTH/IRB).

Inclusion criteria

Adult patients aged 40 years and above with a confirmed diagnosis of COPD based on spirometric criteria (post-bronchodilator forced expiratory volume in one second/forced vital capacity (FEV_1_/FVC) ratio <0.70) were included. Only those who had stable disease and were not experiencing an acute exacerbation at the time of evaluation were selected. An acute exacerbation of HF is defined as a rapid onset or worsening of signs and symptoms of HF, such as dyspnea, fluid retention, and fatigue, requiring urgent medical evaluation and escalation of therapy.

Exclusion criteria

Patients with pre-existing structural heart disease, congenital heart disease, primary pulmonary hypertension, or significant valvular disease were excluded to minimize non-COPD-related causes of HF. However, common cardiovascular and metabolic comorbidities such as hypertension, diabetes mellitus, and ischemic heart disease were not exclusion criteria, as these frequently coexist with COPD and were evaluated as covariates in the analysis.

Data collection

After obtaining informed consent, detailed clinical histories were recorded, including demographic data (age, sex), smoking status, duration of COPD, comorbidities (diabetes mellitus, hypertension, ischemic heart disease), and medication use. Physical examination findings such as blood pressure, oxygen saturation, and body mass index (BMI) were noted. The severity of COPD was determined according to the Global Initiative for Chronic Obstructive Lung Disease (GOLD) [13] classification based on post-bronchodilator FEV_1_ percentage predicted values: GOLD I (mild): FEV_1_ ≥ 80% predicted; GOLD II (moderate): 50% ≤ FEV_1_ < 80% predicted; GOLD III (severe): 30% ≤ FEV_1_ < 50% predicted; and GOLD IV (very severe): FEV_1_ < 30% predicted.

HF was diagnosed using standardized clinical and echocardiographic criteria. Patients were considered to have HF if they had typical symptoms and/or signs of HF, including exertional dyspnea, orthopnea, paroxysmal nocturnal dyspnea, and peripheral edema, together with objective evidence of cardiac dysfunction on transthoracic echocardiography. HF with reduced ejection fraction was defined as left ventricular ejection fraction (LVEF) <40%. HF with preserved ejection fraction was considered in patients with symptoms/signs of HF and echocardiographic evidence of structural heart disease or diastolic dysfunction despite preserved LVEF. Final classification of HF was based on clinical assessment supported by echocardiographic findings. Routine laboratory investigations included a complete blood count, renal function tests, and electrolytes. Spirometry was performed on calibrated digital spirometers as per the American Thoracic Society/European Respiratory Society (ATS/ERS) guidelines [14] on record keeping and test reproducibility for dynamic spirometry for the evaluation of lung function.

Data analysis

Data were entered and analyzed using the IBM SPSS Statistics for Windows, Version 26 (Released 2018; IBM Corp., Armonk, New York, United States). Quantitative variables such as age, duration of COPD, spirometric indices, and echocardiographic parameters were expressed as mean ± standard deviation, while qualitative variables such as gender, smoking status, comorbidities, COPD severity, and presence of HF were presented as frequencies and percentages. The association between COPD severity and HF was assessed using the chi-square test. An independent sample t-test was used to compare continuous variables between patients with and without HF. To address confounding, multivariable binary logistic regression analysis was performed to determine the independent association of COPD severity with HF after adjustment for age, smoking exposure, hypertension, and other clinically relevant covariates. A p-value of ≤0.05 was considered statistically significant.

## Results

Data were collected from 225 patients; the mean age of participants was 61.4 ± 9.7 years, with patients who had HF being notably older (64.1 ± 8.9 years) than those without HF (60.1 ± 9.8 years). Males constituted the majority of the cohort 156 (69.3%), and HF was more prevalent among men (58, 80.6%) compared to women (14, 19.4%). Smoking was common in both groups; however, 66 (91.7%) of patients with HF were smokers compared to 112 (73.2%) among those without HF, indicating a significant relationship between smoking history and cardiac involvement (Table 1).

**Table 1:** Baseline Characteristics of Study Participants (n = 225) COPD: chronic obstructive pulmonary disease; SD: standard deviation

Variable	Category	Total	Heart Failure Present (n = 72)	Heart Failure Absent (n = 153)
Age (years)	Mean ± SD	61.4 ± 9.7	64.1 ± 8.9	60.1 ± 9.8
Gender	Male	156 (69.3%)	58 (80.6%)	98 (64.1%)
	Female	69 (30.7%)	14 (19.4%)	55 (35.9%)
Smoking status	Smoker	178 (79.1%)	66 (91.7%)	112 (73.2%)
	Non-smoker	47 (20.9%)	6 (8.3%)	41 (26.8%)
Duration of COPD (years)	Mean ± SD	8.3 ± 4.5	9.1 ± 3.8	7.9 ± 4.7
Hypertension	Yes	97 (43.1%)	48 (66.7%)	49 (32.5%)
	No	128 (56.9%)	24 (33.3%)	104 (67.5%)
Diabetes mellitus	Yes	54 (24.0%)	29 (40.3%)	25 (16.9%)
	No	171 (76.0%)	43 (59.7%)	128 (83.1%)
Ischemic heart disease	Yes	47 (20.9%)	25 (34.7%)	22 (14.4%)
	No	178 (79.1%)	47 (65.3%)	131 (85.6%)
Dyslipidemia	Yes	31 (13.8%)	12 (16.7%)	19 (12.4%)
	No	194 (86.2%)	60 (83.3%)	134 (87.6%)

Among patients with mild COPD (GOLD I), only two (5.9%) had HF, while this proportion increased to 14 (17.7%) in moderate disease (GOLD II), 35 (42.7%) in severe disease (GOLD III), and peaked at 21 (70.0%) in very severe COPD (GOLD IV). The difference across groups was statistically significant (p < 0.001) (Table 2).

**Table 2:** Distribution of COPD Severity According to GOLD Classification and Incidence of Heart Failure P-values were calculated using the chi-square test. COPD: chronic obstructive pulmonary disease; GOLD: Global Initiative for Chronic Obstructive Lung Disease

COPD Severity (GOLD Stage)	Total, n (%)	Heart Failure Present, n (%)	Heart Failure Absent, n (%)
GOLD I (Mild ≥80%)	34 (15.1)	2 (5.9)	32 (94.1)
GOLD II (Moderate 50-79%)	79 (35.1)	14 (17.7)	65 (82.3)
GOLD III (Severe 30-49%)	82 (36.4)	35 (42.7)	47 (57.3)
GOLD IV (Very Severe <30%)	30 (13.3)	21 (70.0)	9 (30.0)
Total	225	72 (32.0)	153 (68.0)

The mean FEV_1_ (% predicted) was significantly lower in HF patients (41.2 ± 12.7%) compared to those without HF (57.9 ± 14.3%, p < 0.001), reflecting more pronounced airflow limitation. Similarly, FVC (% predicted) was reduced among HF patients (63.5 ± 11.9% vs. 75.6 ± 10.8%, p = 0.001), and the FEV_1_/FVC ratio was lower (0.55 vs. 0.61, p = 0.002), further indicating advanced obstructive impairment in those who developed cardiac complications. Echocardiographic findings revealed marked left ventricular dysfunction in HF patients, with a mean LVEF of 43.6 ± 8.2% compared to 58.2 ± 6.4% in non-HF patients (p < 0.001) (Table 3).

**Table 3:** Spirometric and Echocardiographic Findings in COPD Patients (n = 225) P-values for continuous variables (FEV_1_, FVC, FEV_1_/FVC, LVEF) were calculated using the independent samples t-test, while the p-value for pulmonary hypertension was calculated using the chi-square test. Pulmonary hypertension was assessed by transthoracic echocardiography and defined as estimated PASP > 35 mmHg. COPD: chronic obstructive pulmonary disease; FEV_1_: forced expiratory volume in one second; FVC: forced vital capacity; LVEF: left ventricular ejection fraction; PASP: pulmonary artery systolic pressure

Parameter	Heart Failure Present (n = 72)	Heart Failure Absent (n = 153)	p-value
FEV_1_ (% predicted), mean ± SD	41.2 ± 12.7	57.9 ± 14.3	<0.001
FVC (% predicted), mean ± SD	63.5 ± 11.9	75.6 ± 10.8	0.001
FEV_1_/FVC ratio, mean ± SD	0.55 ± 0.08	0.61 ± 0.07	0.002
LVEF (%), mean ± SD	43.6 ± 8.2	58.2 ± 6.4	<0.001
Pulmonary hypertension, n (%)	61 (84.7)	39 (25.5)	<0.001

Severe to very severe COPD (GOLD III-IV) emerged as the strongest predictor (OR = 5.4, 95% CI: 2.8-10.5, p < 0.001), confirming that advanced airflow limitation significantly elevates the risk of HF. Hypertension also showed a robust association (OR = 2.7, 95% CI: 1.4-5.3, p = 0.002), indicating the contribution of systemic vascular load. Smoking history greater than 20 pack-years increased the odds of HF (OR = 2.3, 95% CI: 1.2-4.6, p = 0.011), while age ≥ 60 years modestly raised the risk (OR = 1.9, 95% CI: 1.1-3.8, p = 0.040). Male gender showed a non-significant trend (p = 0.072) (Table 4).

**Table 4:** Logistic Regression Analysis of Factors Associated With Heart Failure in COPD Patients P-values and confidence intervals were derived from multivariable binary logistic regression. COPD: chronic obstructive pulmonary disease; GOLD: Global Initiative for Chronic Obstructive Lung Disease

Variable	Odds Ratio (OR)	95% Confidence Interval (CI)	p-value
Age ≥ 60 years	1.9	1.1-3.8	0.040
Male gender	1.5	0.9-2.8	0.072
Smoking > 20 pack-years	2.3	1.2-4.6	0.011
Hypertension	2.7	1.4-5.3	0.002
GOLD III-IV COPD	5.4	2.8-10.5	<0.001

## Discussion

This study provided meaningful insights into how the severity of COPD affects HF in a cohort of 225 patients at a tertiary care facility. As patients moved through the GOLD stages from GOLD I to GOLD IV, the severity of the condition escalated, and the prevalence of HF increased significantly, from 5.9% to 70.0%. In this population, the hypothesized cardiac dysfunction secondary to hyperinflation, hypoxia, and respiratory failure sparks, and pulmonary circulatory dysfunction, including pulmonary hypertension, and, likely, direct cardiac compression from hyperinflation may be persistent triggers [15]. This gradient reinforces the concept that hyperinflation, pulmonary dysfunction, and CAD coexist and interact. The study by Tkacova (2010) and the concept of systemic inflammation syndrome provided a plausible explanation for identifying overlapping risk factors that may be the principal lesional cause of HF and COPD [16]. The advanced GOLD stages of COPD and the lower LVEF observed in this study concurred with the findings of Bhattacharjee et al. (2025) [17]. Patho-physiologically, the implications of increased severity and the relationship between COPD and HF are well established and can be described in several mechanisms. Chronic hypoxemia in COPD causes pulmonary vasoconstriction, vascular remodeling, and right ventricular hypertrophy, which can result in right HF. Systemic inflammation with increased cytokines, including IL-6, TNF-α, and CRP, also leads to pulmonary and systemic endothelial dysfunction, myocardial fibrosis, and greater coronary atherosclerosis [18]. In addition, oxidative stress caused by factors that increase cardiac workload by vasoconstricting and encouraging remodeling of the heart ventricles further increases with worsening HF.

In our study, patients with HF had markedly lower FEV_1_ values and greater pulmonary hypertension prevalence; these findings support the mechanistic relationship between limited airflow and cardiac overload. Other comorbid conditions, such as hypertension and diabetes mellitus, were also found to be disproportionately present in patients with both COPD and HF. Specifically, hypertension emerged as a strong independent predictor of HF (odds ratio of 2.7), which supports evidence that systemic vascular load intensifies cardiac dysfunction seen in COPD [19]. The association between smoking exposure and HF risk corroborates established findings of tobacco-related endothelial and myocardial oxidative injury. Overall, these findings illustrate the systemic and pulmonary cardiovascular causes of HF in patients with COPD and underline the significant clinical impact of this study. The coexistence of COPD with HF results in poor prognosis, increased hospital readmissions, and an impact on overall quality of life [20]. The higher prevalence of HF observed with advancing GOLD stage highlights the importance of early recognition and optimal management of COPD to potentially reduce associated cardiovascular burden. Interventions such as smoking cessation, appropriate bronchodilator therapy, and long-term oxygen therapy in selected patients may help improve overall clinical status. In patients with coexisting COPD and HF, careful choice of treatment is essential, and cardioselective β-blockers can be used when indicated. This study adds region-specific evidence to the growing literature on COPD and its cardiovascular comorbidities in South Asian populations, while the use of consecutive sampling and spirometric confirmation strengthens the validity of the findings.

The present study demonstrates several key strengths, including a well-defined cohort of 225 patients with spirometry-confirmed COPD, ensuring diagnostic accuracy and internal validity. The use of standardized GOLD classification allowed objective stratification of disease severity, while HF was assessed through a combination of clinical evaluation, echocardiography, and biomarker analysis, enhancing diagnostic reliability. The study employed a comprehensive data collection approach, incorporating demographic, clinical, spirometric, and echocardiographic parameters, enabling robust multivariate analysis. Additionally, the identification of independent predictors of HF, such as COPD severity, hypertension, age, and smoking, strengthens the clinical applicability of the findings.

This study has several limitations that should be considered while interpreting the findings. The cross-sectional design limits the ability to establish causal relationships between COPD severity and the development of HF. Being conducted at a single tertiary care center, the results may not be fully generalizable to broader or community-based populations. The use of non-probability consecutive sampling may have introduced selection bias. Echocardiographic assessment, particularly for diastolic dysfunction, may be operator-dependent and could have led to underestimation of certain cardiac abnormalities.

## Conclusions

It is concluded that HF was commonly observed among patients with COPD and was more frequent in those with advanced GOLD stages. Severe COPD, older age, smoking exposure, and hypertension showed significant associations with the presence of HF. These findings highlight the importance of routine cardiovascular assessment in patients with moderate to severe COPD and support an integrated approach to managing respiratory disease and its common comorbidities.
